# *De novo* and inherited micro-CNV at 16p13.11 in 21 Chinese patients with defective cardiac left-right patterning

**DOI:** 10.3389/fgene.2024.1458953

**Published:** 2024-09-09

**Authors:** Kun Yu, Weicheng Chen, Yan Chen, Libing Shen, Boxuan Wu, Yuan Zhang, Xiangyu Zhou

**Affiliations:** ^1^ The Fourth Affiliated Hospital of Soochow University, Suzhou Dushu Lake Hospital, Soochow, China; ^2^ Pediatric Cardiovascular Center, Children’s Hospital of Fudan University, Shanghai, China; ^3^ Obstetrics and Gynecology Hospital of Fudan University, Fudan University Shanghai Medical College, Shanghai, China; ^4^ International Human Phenome Institutes (IHPI), Shanghai, China; ^5^ Shanghai First Maternity and Infant Hospital, Tongji University School of Medicine, Shanghai, China

**Keywords:** 16p13.11, copy number variation, deletion, duplication, congenital heart disease, ciliopathy, laterality disorder, left-right patterning

## Abstract

**Objective:**

Copy number changes at Chromosomal 16p13.11 have been implicated in a variety of human diseases including congenital cardiac abnormalities. The clinical correlation of copy number variants (CNVs) in this region with developmental abnormalities remains controversial as most of the patients inherit the duplication from an unaffected parent.

**Methods:**

We performed CNV analysis on 164 patients with defective left-right (LR) patterning based on whole genome-exome sequencing (WG-ES) followed by multiplex ligation-dependent probe amplification (MLPA) validation. Most cases were accompanied with complex congenital heart disease (CHD).

**Results:**

CNVs at 16p13.11 were identified in a total of 21 cases, accounting for 12.80% (21/164) evaluated cases. We observed a marked overrepresentation of chromosome 16p13.11 duplications in cases when compared with healthy controls according to literature reports (15/164, 9.14% versus 0.09% in controls). Notably, in two independent family trios, *de novo* 16p13.11 micro-duplications were identified in two patients with laterality defects and CHD. Moreover, 16p13.11 micro-duplication was segregated with the disease in a family trio containing 2 affected individuals. Notably, five coding genes, NOMO1, PKD1P3, NPIPA1, PDXDC1, and NTAN1, were potentially affected by micro-CNV at 16p13.11 in these patients.

**Conclusion:**

Our study provides new family-trio based evidences to support 16p13.11 micro-duplications predispose individuals to defective cardiac left-right patterning and laterality disorder.

## Introduction

Abnormalities in the ultrastructure of cilia result in a rapidly expanding spectrum of clinical symptoms, termed ciliopathies, that mainly include nephronophthisis, Bardet–Biedl syndrome, primary ciliary dyskinesia (PCD), and retinal degeneration, laterality disorder (Fliegauf et al., 2007; Reiter and Leroux, 2017). Classified by microtubular structure, two types of cilia are found: primary/non-motile cilia and regular motile cilia. Most regular motile cilia consist of a ring of nine peripheral microtubule doublets that surrounds a central pair of single microtubules (9 + 2 structure). In contrast to the 9 + 2 pattern of regular cilia, primary or sensory cilia are solitary, immotile organelles that have 9 + 0 microtubule configuration and are present on most cell types.

Proper left-right (LR) asymmetry is an essential aspect of embryonic development (Hamada et al., 2002). Embryonic cilia-driven fluid flow plays essential roles in LR organization in mammals (Hirokawa et al., 2006). Defects in LR organization cause a range of laterality disorders including situs inversus (SI) totalis (SIT) and heterotaxy (Htx) (Sutherland and Ware, 2009). SIT is a congenital condition in which organs in the chest and abdomen are arranged in a complete mirror-image reversal of the usual positions; the prevalence of SIT is estimated to range from 1/25,000 to 1/8,000 (Bartoloni et al., 2002). Up to 20% of SIT patients have Kartagener syndrome (KS), which is a triad of nasal polyps, bronchiectasis, and SIT, and a subgroup of PCD (Leigh et al., 2009). Approximately, 50% of patients with PCD present with SIT. Htx is a condition that involves the internal organs being abnormally arranged. However, unlike SIT, it does not result in a mirror-image reversal of organ positions. Over 80% of individuals with Htx present with complex CHDs including transposition of the great arteries (TGA) and double outlet right ventricle (DORV) (Fakhro et al., 2011). SIT patients have a lower risk of CHD (3%–9%) than Htx patients (Taketazu et al., 2006). However, the risk of CHD in SIT is still significantly higher than that in the normal condition, situs solitus (0.6%–0.8%) (Peeters and Devriendt, 2006). Till date, dozens of candidate genes including *DNAH5, DNAH9, TTC21B, NUP205, NEK3, NPHP3, CCDC40* and *NEK8* have been reported (Reiter and Leroux, 2017; Nöthe-Menchen et al., 2019; Chen et al., 2022; Zhang et al., 2020; Chen et al., 2019).

Copy number changes at chromosomal 16p13.11 have been recently implicated in pseudoxanthoma elasticum (van Soest et al., 1997), a variety of neuropsychiatric disorders (Johnstone et al., 2019), congenital anomalies of the kidney and urinary tract (CAKUT) (Verbitsky et al., 2019), megacystis-microcolon-intestinal-hypoperistalsis syndrome (MMIHS) (Kloth et al., 2019) and congenital cardiac abnormalities (Allach El Khattabi et al., 2018; Hamad et al., 2023). The population frequency of 16p13.11 duplication is estimated to be 0.09% in controls (Ingason et al., 2011; Kuang et al., 2011), 0.30% in schizophrenia (Ingason et al., 2011), 1.04% in aortic dissections (Kuang et al., 2011), and approximately 0.20% in CAKUT (Verbitsky et al., 2019). Although genotype-phenotype analysis does not reveal the impact of the size of duplicated segments on the severity of the phenotype, ciliary-related nuclear distribution E (NDE1) was proposed to be a response for the neuropsychiatric phenotype (Heinzen et al., 2010). In addition, modulators of Nodal, Nomo1 and Nomo3, were presented in this region (Warburton et al., 2014). These findings suggested that 16p13.11 alterations might be involved in the normal biological processes underlying ciliary function.

## Methods

### Study participants

Individuals with LR pattering defects were diagnosed by X-ray and color ultrasonic diagnosis at Pediatric Cardiovascular Center of the Children’s Hospital affiliated to Fudan University. Typical symptoms of PCD, including neonatal rhinosinusitis, airway infections, and ottis media, were determined by standard clinical diagnostic criteria and nasal nitric oxide measurements as previously described (Chen et al., 2022). Other malformations in the cardiovascular system were diagnosed by cardiac ultrasound. For studies of affected individuals and their families, written informed consent was obtained from all participants prior to the start of the study. All procedures in the study were approved by the Medical Ethics Committee of Children’ Hospital of Fudan University (2016-079) (Shanghai, China).

### WE-GS

Genomic DNA isolated from the peripheral blood of patients was used to perform whole genome or exome sequencing on the SureSelect human all exon platform (v.6; Agilent Technologies, Santa Clara, CA, United States). Exome-enriched genomes were multiplexed using a flow cell for paired-end 2 × 150-bp read sequencing based on protocols established for the HiSeq X10 platform (Illumina, San Diego, CA, United States). The Genome Analysis Toolkit software package was used to detect single-nucleotide variants and indels. On average, exome coverage and depth were more than 95% (depth >20) and 80×, respectively.

### CNV analysis

All sequencing data in this study were trimmed and filtered with Fastp. The filtered sequencing reads were aligned to a hg19 reference genome (human_g1k_v37.fasta) with Burrows-Wheeler Aligner (bwa 0.7.16,http://bio-bwa.Sourceforge.net/) and the duplicates were removed with samtools markdup (samtools 1.9). Then the Genome Analysis Toolkit (gatk 4.0.12.0) was employed for base quality score recalibration and indel realignment. The common genetic variants were recalibrated using GATK Resource Bundle (dbSNP, HapMap, g1k snps and indels). We applied the popular somatic copy number alteration caller, VarScan (VarScan v2.3.9), to the pre-processed sequencing data with the workflow recommended by developers. The called copy numbers with Varscan between two samples were smoothed and segmented using the R library and DNAcopy.

### Multiplex-ligation dependent probe amplification (MLPA)

MLPA was used to validate the specific small chromosomal abnormalities at 16p13.11 identified from CNV analysis based on WE-GS data by following standard protocol. Three different probes were designed for each targeted gene (*GAPDH* and *PDXDC1*). The sequences of probes were provided in Supplementary Table S1. GAPDH served as internal reference. Copy number status was determined based on the DQ (dosage quotient) values (0.80<DQ < 1.20, Normal; DQ = 0.00, homozygous deletion; 0.40<DQ < 0.65, heterozygous deletion; 1.3<DQ < 1.65, heterozygous duplication; 1.75<DQ < 2.15, heterozygous triplication/homozygous.

### Statistical analysis

Here we compared the prevalence of duplication at 16p13.11 in our studied cohort with the prevalence in a control population and thoracic aortic aneurysm and dissection (TAAD) cohort from two previous studies. We calculated statistical significance using the Chi-square test (χ2). A *p*-value that is less than or equal to the 0.05 significance level signifies statistical significance.

## Results

**FIGURE 1:**
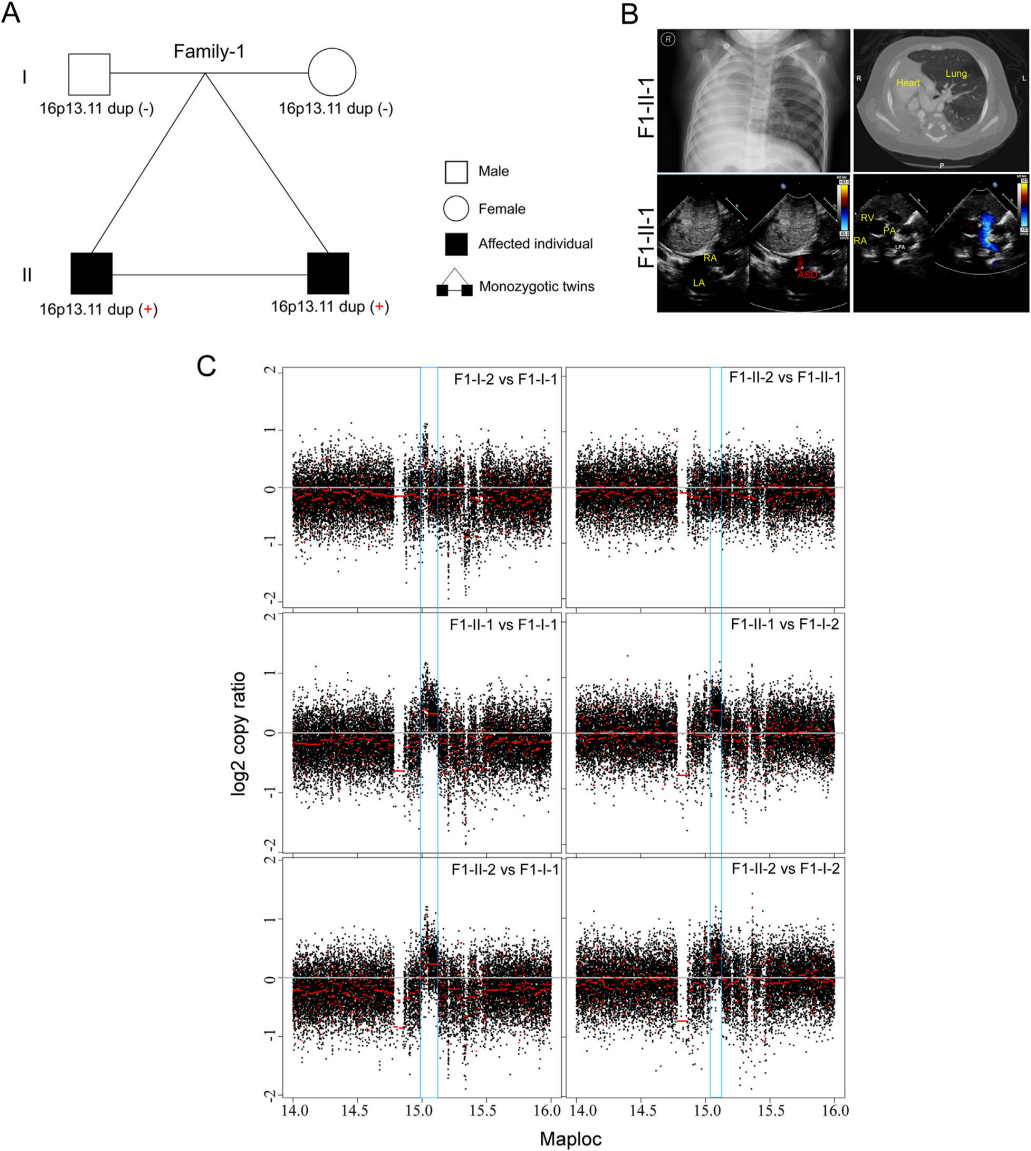
*De novo* 16p13.11 duplication in a monozygotic twin **(A)** Pedigrees of Family-1 (F1) indicating the affected individuals and the distribution of 16p13.11 duplication. **(B)** Chest X-ray and CT-scan show isolated dextrocardia in the proband (F1-II-1) (upper), and color ultrasound scans reveal an atrial septal defect (ASD), and a pulmonary artery sling in the patient (bottom). RA, right atrium; LA, left atrium; RV, left ventricle; LPA, left pulmonary artery; PA, pulmonary artery. **(C)** CNV analysis based on WGS data identified *de novo* 16p13.11 micro-duplication (chr16: 14.98–15.13, 150 kb, GRCh37.p13) in both sibilings when compared to the parents, independently.

In this study, we initially recruited three unrelated family trios with laterality defects and CHD (Table 1; Figure 1A). In a monozygotic twin (Family-1), a 4-year-old boy (F1-II-1) displayed isolated dextrocardia accompanied by an absent right lung, an atrial septal defect, and a pulmonary artery sling (Figure 1B). The identical twin brother (F1-II-2) exhibited congenital nystagmus and already undergo ophthalmic surgery, without other developmental malformations. Through trio-based whole genome sequencing (WGS) analysis on four family members, we did not identify recessive mutations in well-known ciliary genes that could meet our filter strategy. Subsequent genome-wide CNV analysis then identified *de novo* 16p13.11 micro-duplication (chr16: 14.98–15.13, 140 kb) in both affected siblings (Figure 1C). Five coding genes, NOMO1 (NODAL modulator 1) (chr16:14927578-14990014), PKD1P3 (polycystin 1, transient receptor potential channel interacting pseudogene 3) (chr16:15011391-15029565), NPIPA1 (nuclear pore complex interacting protein family member A1) (chr16: 15.03–15.05 Mb), PDXDC1 (pyridoxal dependent decarboxylase domain containing 1) (chr16:15.07–15.12 Mb), NTAN1 (N-terminal asparagine amidase) (chr16:15131714-15149931) were potentially affected by the duplicated region.

**TABLE 1:** Clinical characteristics of 21 patients (12 males, nine females) with 16p13.11 copy number changes. All 21 patients displayed defective cardiac LR patterning, mainly SIT and dextrocardia. The majority (17/21) of these individuals present with complications including congenital heart disease (10/21), PCD (3/21), kidney disease (2/21), congenital anal atresia (1/21) and congenital nystagmus (1/21). *De novo* copy number changes were identified in 33.3% (7/21) individuals.Three patients harbor the second rare CNV at 16p11.2. M, male; F, female; Dup, duplication; Del, deletion; n.a, not available; PCD, primary ciliary dyskinesia; SIT, situs inversus (SI) totalis; VSD, ventricular septal defect.

Patients	Age	Gender	Defective LR patterning	Complications	16p13.11		Second CNV
No. 1	4	M	Isolated dextrocardia	VSD and pulmonary artery sling	Dup	*De novo*	—
No. 2	4	M	—	Congenital nystagmus	Dup	*De novo*	—
No. 3	26	M	SIT	—	Dup	Inherited	—
No. 4	51	F	SIT	—	Dup	Inherited	—
No. 5	6	M	SIT	Nephronophthisis	Dup	*De novo*	16p11.2 deletion
	3	F	—	Neonatal cholestasis, chronic renal disease	—	n.a	—
No. 6	5	F	Dextrocardia	Recurrent respiratory infection, pulmonary agenesis	Dup	*De novo*	—
No. 7	5	M	Dextrocardia	Atrial septal defect, Persistent Left Superior Vena Cava	Dup	Inherited	—
No. 8	7	M	Isolated dextrocardia	Persistent Left Superior Vena Cava	Dup	Inherited	—
No. 9	2	M	SIT	Bicuspid aortic valve	Dup	n.a	—
No.10	2	M	Dextrocardia	VSD, Biliary atresia, Ectopic kidney	Dup	n.a	—
No. 11	1	F	Dextrocardia	VSD, Double outlet right ventricle	Dup	inherited	—
No. 12	6	F	Dextrocardia	Complete transposition of great arteries, Asplenia	Del	*De novo*	—
No. 13	12	F	SIT	Patent foramen ovale, VSD	Del	*De novo*	—
No. 14	6	M	SIT	—	Del	Inherited	—
No. 15	5	F	Dextrocardia	Double outlet right ventricle, Patent ductus arteriosus	Del	*De novo*	—
No. 16	6	M	SIT	PCD	Del	Inherited	16p11.2 deletion
No. 17	4	F	SIT	—	Dup	Inherited	—
No. 18	8	M	SIT	Congenital anal atresia	Del	Inherited	—
No. 19	9	F	SIT	PCD	Dup	n.a	—
No. 20	3	M	SIT	VSD, Atrial septal defect	Dup	Inherited	16p11.2 deletion
No. 21	5	F	Dextrocardia	Transposition of great arteries, VSD	Dup	n.a	—

**FIGURE 2:**
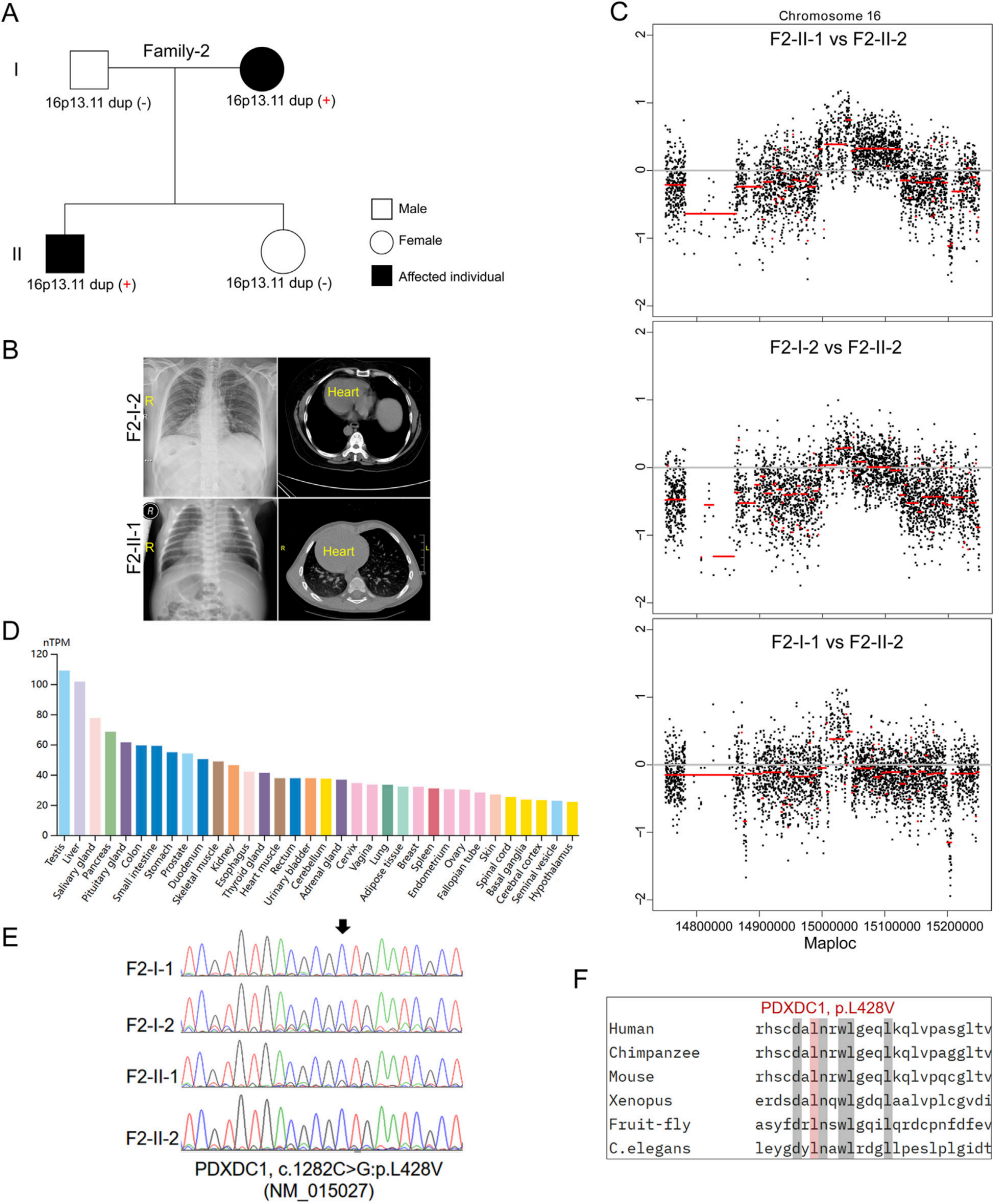
Inherited 16p13.11 duplication segregated with the disease in Family-2. **(A)** Pedigrees of Family-2 (F2) indicating the affected individuals and the distribution of 16p13.11 duplication. **(B)** Representative images of chest X-ray and CT scan shows the mirror-image arrangement of the abdominal organs in both patients (F2-I-2 and F2-II-1), R, right. **(C)** CNV analysis based on WGS data identified 16p13.11 micro-duplication (chr16: 14.98–15.13, GRCh37.p13) in both patients when compared to the unaffected individual, respectively. **(D)** Human Protein Atlas database showing PDXDC1 is widely expressed in various human tissues and has highest levels in testis. **(E)** Sanger sequencing on PDXDC1 variant (p.L428V) in the patients and unaffected members. **(F)** Sequencing alignment of missense variants p.L428V in different species as indicated. **(C)** elegans, *caenorhabditis elegans*.

In the second family (Family-2) trio containing 2 affected individuals with SIT and 2 unaffected individuals (Figures 2A, B). The proband (F2-II-1) was a 26-year-old male without detectable complications during his life history to the present (chief complaint but sperm morphological examination is not included). His mother (F2-I-2) is a 51-year-old woman and her typical PCD phenotypes including bronchitis, sinusitis, and otitis media, whom were excluded by physical examination as previously (Chen et al., 2022). Echocardiography did not reveal other detectable abnormalities in heart structure or motion in both individuals. It is uncommon that SIT occurs in an autosomal dominant pattern in a non-consanguineous marriage family. However, we did not identify potential causative recessive and sex-linked non-synonymous variants in the known candidate genes by trio-based WGS and WES. Subsequent CNV analysis found that micro-duplication at 16p13.11 was identified in both patients (F2-II-1 and F2-I-2) (Figure 2C), but not in two unaffected members, suggesting that 16p13.11 duplication might segregated with the disease in this family. Moreover, WGS analysis identified a heterozygous missense mutation in PDXDC1 (c.1282C>G, p.L428V, NM_015027) in both affected individuals. PDXDC1 is widely expressed in various human tissues and has highest mRNA level in testis (Figure 2D), which is a common feature of many cilia related genes. Sanger sequencing further validated the base substitution from C to G at c.1282 of PDXDC1 in the patients (Figure 2E). The CADD_Phred and GERP++_RS score of PDXDC1 L428V is 11.72 and 2.90, respectively. Although sequence alignment indicated PDXDC1 L428V affected highly conserved residues across including *Xenopus* tropicalis and *Caenorhabditis elegans* (Figure 2F), allele frequency of PDXDC1 p.L428V in GnomAD (v2.1.1)_exome_all (MAF = 0.0353) and gnomAD_exome_EAS (MAF = 0.0417) is too high for a disease-causing factor. Therefore, we classify L428V as a VUS (Variant of Uncertain Significance) leaning towards likely benign if applying the ACMG (American College of Medical Genetics and Genomics) guidelines.

**FIGURE 3:**
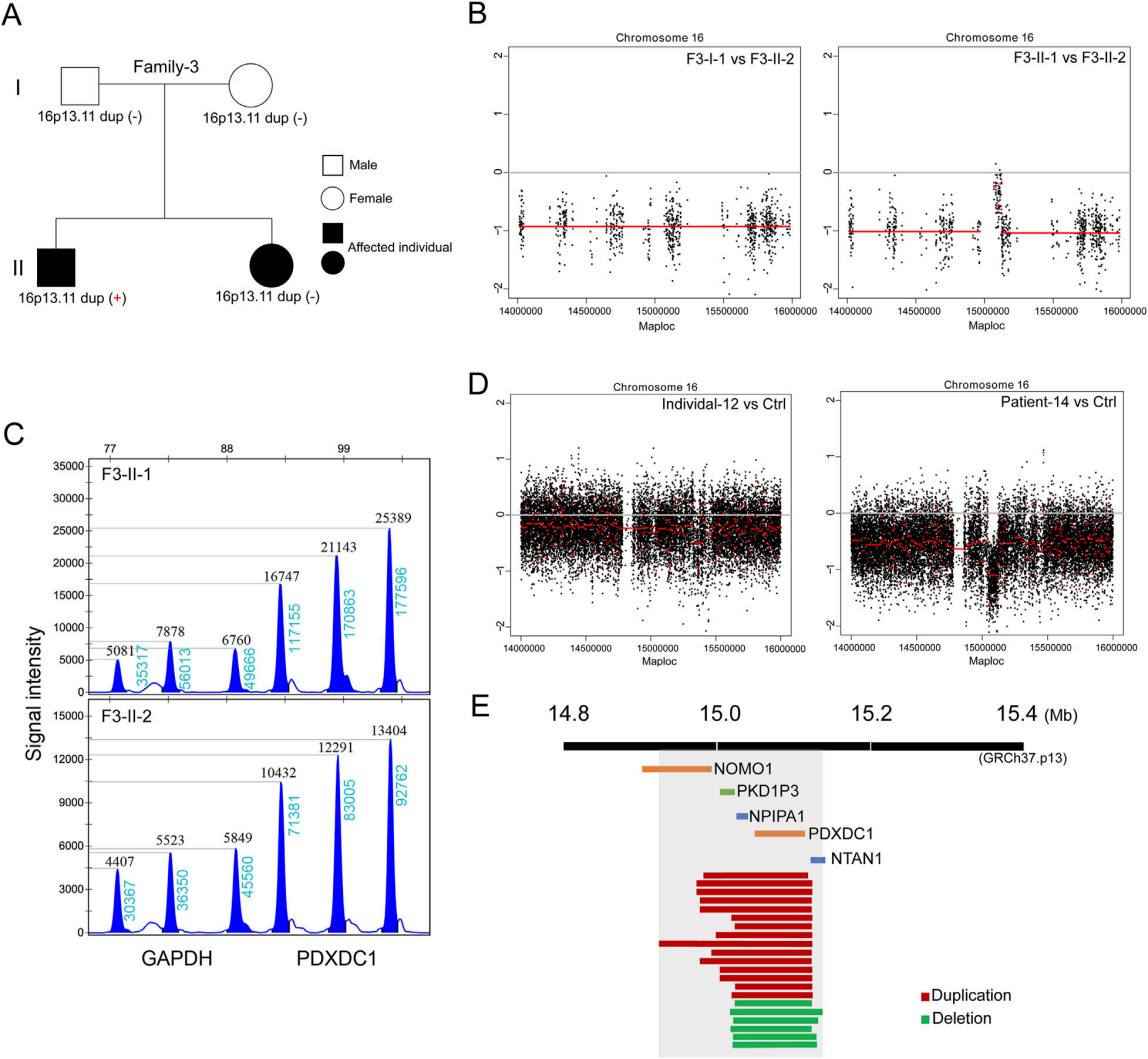
*De novo* 16p13.11 duplication potentially modulates the cardiac phenotype. **(A)** Pedigrees of Family-2 (F3) indicating the affected individuals and the distribution of 16p13.11 duplication in two siblings with different ciliopathies. **(B)** CNV analysis based on WES data identified micro-duplication at 16p13.11 in the brother (F3-II-1) but not in the sister (F3-II-2). **(C)** MLPA confirmation of the gain of copy in *PDXDC1*, located in the 16p13.11 region, that identified from CNV analysis in the F3-II-1 when compared with F3-II-2. Three independent probes were designed for each targeted gene as indicated. GAPDH served as the internal reference. Dosage quotient (DQ) values = 1.501 (1.3<DQ < 1.65, heterozygous duplication) **(D)** Genome-wide CNV analysis also identified 16p13.11 deletion in the patient with defective LR patterning when compared with health Ctrl (right panel). Left panel served as a internal Ctrl. **(E)** The diagram of 16p13.11 duplications (red) and deletions (green) in 21 patients. The scale is in megabases. The region that is spanned by all CNVs containing five coding genes, *NOMO1, PKD1P3, NPIPA1, PDXDC1 and NTAN1,* as indicated.

In the third family (Family-3) trio containing two affected siblings (Figure 3A). The proband is a 6-year-old boy (F3-II-1) diagnosed with nephronophthisis-related ciliopathies and SIT. The patient’s 3-year-old sister (F3-II-2) exhibited severe neonatal cholestasis and chronic renal disease, but had no defects in heart development. Subsequent trio-based WES did not identify the well-known causative genes in both siblings, according to literature reports (Reiter and Leroux, 2017). Genome-wide CNV analysis then identified *de novo* 16p13.11 duplication in the proband but not in his sister (Figures 3B, C). This finding further support that 16p13.11 duplication might act as a modifier of phenotypic heterogeneity on cardiac LR patterning.

Based on our above-mentioned findings, we expand the clinical sample size and performed CNV analysis on patients with defective cardiac LR patterning based on WG-ES data to verify the main findings above. A total of 164 cases (median age: 3.25 years; range: 35 days-51 years) confirmed to have laterality disorders were recruited between January 2013 and July 2023. Associated conditions were diagnosed in 139 cases (84.75%). The most commonly associated conditions were congenital heart defects (n = 116, 70.73%) followed by primary ciliary dyskinesia (n = 15, 9.15%) and renal disorders (n = 6, 3.66%). We identified copy number changes at 16p13.11 in a total of 21 patients (Table 1; Supplementary Figure S1), 15 cases with 16p13.11 micro-duplication (15/164, 9.14%) and 6 cases with 16p13.11 micro-deletion (6/164, 3.65%) (Figure 3D). We found that the frequency of 16p13.11 duplication in laterality disorders (9.14%) was markedly higher than that in controls (0.09%) and other relative disease as aforementioned (Verbitsky et al., 2019; Ingason et al., 2011; Kuang et al., 2011), ranging from 0.3% to 1.04%. The common region (chr16: 15.02–15.13, 110 kb, GRCh37.p13) that is spanned by all CNVs in 21 patients containing two intact coding genes (Figure 3E), NPIPA1 and PDXDC1 as aforementioned. Furthermore, three coding genes NOMO1, PKD1P3 and NTAN1 were also potentially, at least in part, affected by these micro-CNVs at 16p13.11.

## Discussion

Here we report a significant overrepresentation of chromosome 16p13.11 duplications in patients with defects in cardiac LR patterning (14.42% versus 0.09% in controls as previously) (Ingason et al., 2011; Kuang et al., 2011), indicating greater enrichment of this duplication in laterality disorders. Cardiac malformations were present in approximately 20% of patients carrying 16p13.11 duplications, indicating a significant risk of cardiovascular disease (Allach El Khattabi et al., 2018; Nagamani et al., 2011). In 2023, a multicentric analysis of 206 patients with 16p13.11 microduplication. Echocardiograms were performed in 50.5% (104/206) of the total patient cohort and a congenital cardiac anomaly was identified in 15% (16/104) of those patients (Hamad et al., 2023). Duplication of the distal 16p13.11 recurrent region has been associated with variable clinical phenotypes including developmental delay, intellectual disability, learning difficulties, behavioral abnormalities, and variable dysmorphic features (Verbitsky et al., 2019). Although primary cilia play the important roles in congenital heart defect-associated neurological impairments, none of 21 patients in our study exhibited these neuropsychiatric disorders to date. We understand that neurological phenotypes in these CHD cases might be ignored due to the relative poor quality of medical conditions in grass roots. Ciliary-related NDE1 was consistently included in the 16p13.11 deletions in 23 previously evaluated patients with epilepsy syndromes (Ingason et al., 2011) and eight patients with TAAD (Chen et al., 2022). However, we did not detect either gained or lost copies of NDE1 in our 21 patients, which raises the possibility of other pathogenic genes responding in non-neuropsychiatric phenotypes.

Five coding genes, NOMO1, PKD1P3, NPIPA1, PDXDC1 and NTAN1, were potentially affected by 16p13.11 alterations in these patients. NOMO1 was identified as part of a protein complex that participates in the Nodal signaling pathway during vertebrate development. NODAL flow plays essential role in the generation of left-right asymmetry. Overexpression of NOMO1 imposes a sheet morphology on the endoplasmic reticulum (Amaya et al., 2021). Nomo1-deficient zebrafish exhibit multiple neuropsychiatric behaviors such as hyperactive locomotor activity, social deficits, and repetitive stereotypic behaviors (Zhang et al., 2024). NPIPA1 could co-localize with cilia-related NUP62 (Johnson et al., 2001; Takao et al., 2017). Many components of nuclear pore complex (NPC), especially inner ring nucleoporins NUP93, NUP205 and NUP188, as well as NUP62 were involved in the ciliary function and LR determination (Chen et al., 2019; Chen et al., 2023; Del Viso et al., 2016; Kee et al., 2012; Marquez et al., 2021). PDXDC1 gene was frequently deleted in hearing loss patients and modulates prepulse inhibition of acoustic startle in the mouse (Feldcamp et al., 2017; Haraksingh et al., 2014). Interestingly, many PCD patients exhibited conductive hearing loss (Kreicher et al., 2018). Although PDXDC1 is not associated with any human disease in OMIM, this gene has highest mRNA level in testis and is conserved in different species. Interestingly, many ciliary genes including NPHP4, CCDC40, DNAH9 and RSPH6A have restricted expression toward testis, suggesting PDXDC1 might be involved in ciliary function. NTAN1 acts as a tertiary destabilizing enzyme that deamidates N-terminal L-Asn residues on proteins to produce N-terminal L-Asp. L-Asp substrates are subsequently conjugated to L-Arg, which is recognized by specific E3 ubiquitin ligases and targeted to the proteasome. The Ntan1 gene is expressed in perineural glia and neurons of adult *Drosophila* (Castañeda-Sampedro et al., 2022). Altered activity, social behavior, and spatial memory was detected in Ntan1 (−/−) mice (Kwon et al., 2000).

Previous study reported that 9.7% of patients carrying 16p13.11 microduplication were found to have a second genetic/chromosomal diagnosis, especially where there were additional phenotypic features (Hamad et al., 2023). In this study, we found that three patients with 16p13.11 duplication were likely to harbor the second rare CNV at 16p11.2. For example, the patient in family-3 (F3-II-1) harbors a second rare CNV in 16p11.2 (Supplementary Figure S2). A recent study found that the rare 16p11.2 deletion could push the genetic background closer to the threshold for severe manifestation and therefore require a lesser contribution from other hits. Thus, accurate genetic diagnosis requires complete evaluation of the genetic background even after a candidate disease-associated variant is identified (Pizzo et al., 2019). This model might explain the phenotypic heterogeneity of disease-associated variants in different siblings.

One limitation of our study is that we do not evaluate the frequency of 16p13.11 micro-duplication in the ethnic matched controls, although the frequency of the 16p13.11 duplication was reported to be identical between several previous studies. Our main concern is the marked phenotypic heterogeneity of 16p13.11 CNVs that were associated with a variety of developmental diseases in human. In this study, most of the patients inherit the duplication from an unaffected parent, with maternal and paternal inheritance evident. These findings further support the hypothesis of incomplete penetrance and that imprinting does not seem to take effect in manifestations, as the micro-duplication can be inherited from either parent (Hamad et al., 2023; Nagamani et al., 2011). Investigating the roles of five affected candidate genes on ciliary function and cardiac LR patterning would be a potential avenue for further understanding of these observations. Despite being a preliminary evaluation, our study indicated that micro-CNV at 16p13.11 predispose individuals to defective establishment of cardiac LR patterning.

## Data Availability

The original contributions presented in the study are included in the article/Supplementary Material, further inquiries can be directed to the corresponding author.
