# Supplementary material for: De novo and inherited micro-CNV at 16p13.11 in 21 Chinese patients with defective cardiac left-right patterning
Source: Front Genet. 2024 Sep 9;15:1458953. doi: 10.3389/fgene.2024.1458953 (PMC11416941; doi:10.3389/fgene.2024.1458953)
Supplement: Supplementary file 1 [file DataSheet1.pdf]

Supplementary Figures

Figure S1. CNV analysis based on WES or WGS data identified 16p13.11 copy number variations in 21 patients with defective cardiac LR patterning. Representative images are displayed as indicated.

Figure S2. CNV analysis indicated that the patient in family-3 (F3-II-1) harbors a second rare CNV in 16p11.2.

Fig.S1

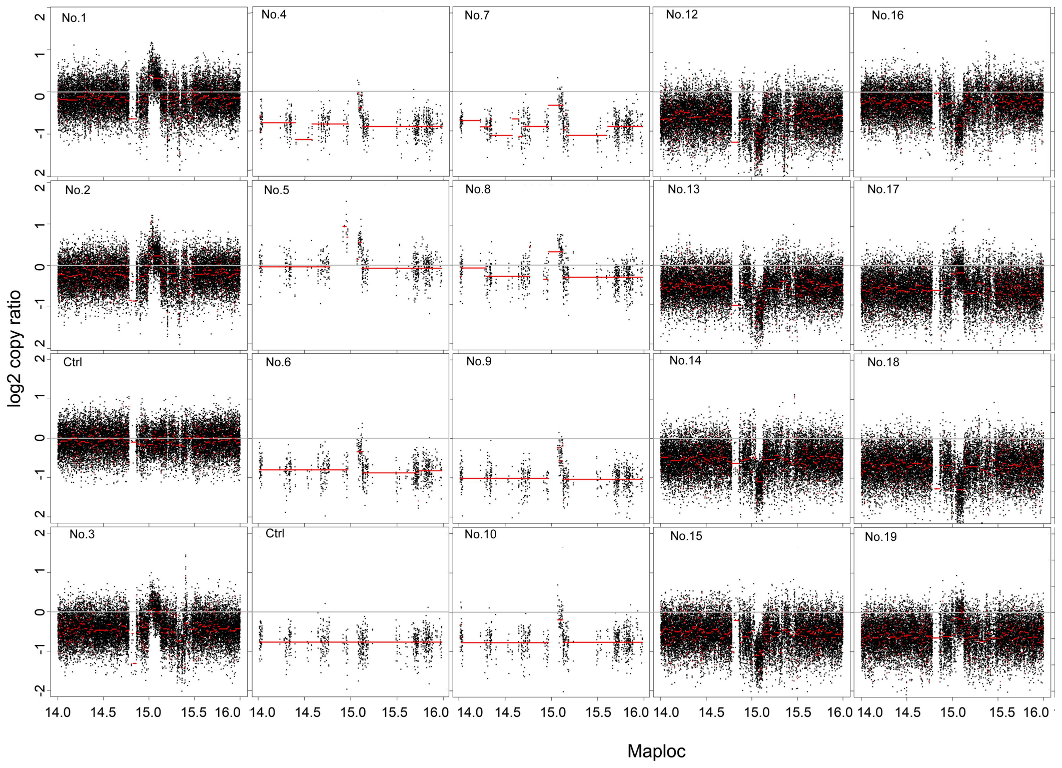
No.1
No.4
No.7
No.12
No.16
No.2
No.5
No.8
No.13
No.17
Ctrl
No.6
No.9
No.14
No.18
No.3
Ctrl
No.10
No.15
No.19
log2 copy ratio
Maploc
14.0 14.5 15.0 15.5 16.0

Fig. S2

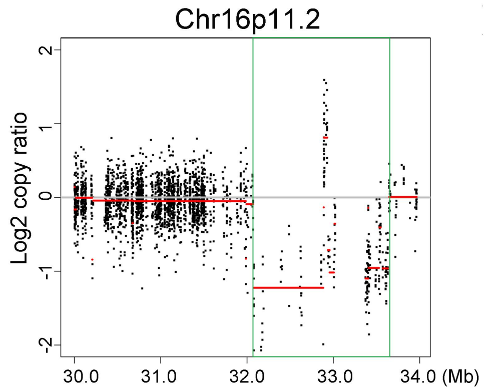
Chr16p11.2
Log2 copy ratio
30.0 31.0 32.0 33.0 34.0 (Mb)
